# Capacitive-Coupling Impedance Spectroscopy Using a Non-Sinusoidal Oscillator and Discrete-Time Fourier Transform: An Introductory Study

**DOI:** 10.3390/s20216392

**Published:** 2020-11-09

**Authors:** Tomiharu Yamaguchi, Akinori Ueno

**Affiliations:** Department of Electrical and Electronic Engineering, Tokyo Denki University, Tokyo 120-8551, Japan; 21769@ms.dendai.ac.jp

**Keywords:** capacitive coupling, impedance spectroscopy, non-sinusoidal oscillator, DFT

## Abstract

In this study, we propose a new short-time impedance spectroscopy method with the following three features: (1) A frequency spectrum of complex impedance for the measured object can be obtained even when the measuring electrodes are capacitively coupled with the object and the precise capacitance of the coupling is unknown; (2) the spectrum can be obtained from only one cycle of the non-sinusoidal oscillation waveform without sweeping the oscillation frequency; and (3) a front-end measuring circuit can be built, simply and cheaply, without the need for a digital-to-analog (D-A) converter to synthesize elaborate waveforms comprising multiple frequencies. We built the measurement circuit using the proposed method and then measured the complex impedance spectra of 18 resistive elements connected in series with one of three respective capacitive couplings. With this method, each element’s resistance and each coupling’s capacitance were estimated independently and compared with their nominal values. When the coupling capacitance was set to 10 nF or 1.0 nF, estimated errors for the resistive elements in the range of 2.0–10.0 kΩ were less than 5%.

## 1. Introduction

Impedance spectroscopy (IS) measures the impedance of targets at various frequencies and assesses their electrical characteristics using spectral analysis. IS is a widely used technique in electrochemistry and biomedical engineering.

In electrochemistry, IS is known as electrochemical impedance spectroscopy (EIS). EIS is used to analyze the characteristics of fuel cells, lithium-ion batteries, and photovoltaic cells [1,2,3,4,5]. It is often used to analyze the response mechanism of chemical sensors and detect response signals in them [6,7,8]. In general, the ionic conduction speed differs in electrode interfaces and the electrolytes of fuel cells and lithium-ion batteries. The former responds to electric fields more slowly than the latter. Therefore, when the frequency of an electric field applied to a measured object is high, the ionic conduction of the electrode interface cannot follow the changes in the alternating field, and, for the most part, there is a bulk impedance component with a fast response speed. Therefore, by measuring the impedance spectrum, each ionic conduction process can be analyzed.

EIS is commonly used to evaluate anti-corrosion properties of metals and to monitor corrosion processes because the corrosion reactions of metals change electrical potential and resistance [9,10]. In addition, it is also applied to the degradation monitoring of anti-corrosive organic coatings [11,12,13,14,15,16,17]. The coatings are degraded or delaminated by corrosion reactions at metal-coating interfaces, which changes their circuit parameters, such as coating resistance and coating capacitance. The coating resistance of anti-corrosive coatings is extremely high, but gradually decreases with their degradation. The coating capacitance depends on the water volume fraction of the coatings [18]. Because most coatings are relatively thick and have low coating capacitance, systems for measuring low capacitance are required for degradation monitoring.

In biomedical engineering, IS is known as bioimpedance spectroscopy (BIS) [19,20,21,22,23]. When measuring impedance for a few frequencies, it is also referred to as bioelectrical impedance analysis (BIA). In a living body, impedance consists of a resistance component dependent on the body’s water content and a capacity component that depends on cell membranes’ capacitance. The body’s composition information, such as its water content, obtained from impedance, is an important indicator of health conditions and their diagnoses. The body’s water content is generally inversely proportional to resistance; thus, low-resistance reflects a high water content in the body. Furthermore, a lower capacitance indicates that there are either not many biological tissues or a high volume of extracellular fluid. Both the BIS and BIA methods can non-invasively determine body compositions and pathologies at relatively low cost; thus, the technique can be used to monitor body fat and body composition [24,25,26].

The non-invasive assessment of body composition is useful for patients with diseases such as heart failure, chronic kidney disease, and cardiorenal syndrome [27]. For a more detailed analysis of body composition in these patients, bioelectrical impedance vector analysis (BIVA) has been proposed [28]. In the BIVA approach, the resistance component R of impedance and reactance component $X_{C}$ are standardized with body height, and the vector of impedance is plotted on an R–$X_{C}$ graph. The absolute value of the vector and its phases are then correlated with tissue hydration and body cell mass in soft tissues, respectively. BIVA is also used to analyze the body composition of patients with cancer [29,30], those undergoing dialysis [31,32,33], those with edema [34,35], and those with polyneuropathy, organomegaly, endocrinopathy, M-protein, and skin change syndromes [36].

BIA is also known as impedance microbiology (IM). The impedance of the growth medium for microorganisms depends on microbial concentrations [37]. When the concentration of microorganisms increases, the proteins, carbohydrates, and fats decompose into amino acids, sugars, etc. Because the medium’s impedance is considered a series connection of the medium’s resistance and the electric double layer capacitance formed between the medium and the electrodes, the ionic and polar substances produced change the medium’s impedance. IM has a range of uses and is applied, for instance, to determine microbial concentrations in ice cream [38] and beer [39].

BIS- and BIA-based sensors for detecting biological substances and agents, such as bladder cancer [40], DNA [41], protein [42], and bacteria [43], have also been studied. These sensors can transduce the interaction of antibodies with their object into an electrical signal.

In food engineering, IS is applied to assess the ripeness of fruits and the freshness of fish, with analytical investigations presented in the literature of apples, bananas, and oranges [44,45], in addition to carp and herring [46].

However, in all fields, measured objects are generally bound with a resistive coupling, and sine waves are applied; thus, IS has rarely been applied to the analysis of insulator–electrolyte interfaces or the measurement of the electrical characteristics of resistive elements with an insulating coating.

**Figure 1:**
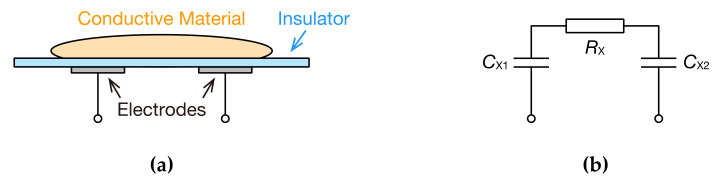
Schematic model of electrodes capacitively coupled to a conductive material via a thin insulator: (**a**) cross-sectional diagram; (**b**) equivalent circuit.

When electrodes come into contact with thin insulating material (Figure 1a), two capacitive couplings form between the resistive elements of the measured object, which can be expressed with an equivalent circuit (Figure 1b). In such a capacitive-coupling state, if the resistive value can be measured in some IS approach, the scope of application of IS will broaden. Furthermore, because IS measures the impedance of multiple frequencies, sine wave signals with different frequencies are input, and responses are measured. Therefore, IS requires a complex circuit of oscillators that can generate multiple sine wave signals. In addition, measurement times increase because multiple signals are sequentially input. To resolve these two problems, a method has been developed in which signals with multiple frequency components are applied to the measured objects, and impedance is determined using a discrete Fourier transform (DFT) of the obtained signals, a fast Fourier transform (FFT), or a short-time Fourier transform (STFT) [47,48,49,50]. These methods are applied to a wide range of fields, such as lithium ion batteries [51,52], corrosion studies [53], materials [54,55], freezing processes of crops [56], and gene detection [57]; however, no reports of capacitive coupling have been published.

In this study, we demonstrate a new short-time capacitive-coupling IS based on the following ideas:

(1) By coupling electrodes capacitively to the measured object and by incorporating the resulting couplings into an oscillation circuit, an alternating current is applicable inside the object covered with a thin insulating layer.
(2) By measuring the amplitude and phase of the object’s current and those of the object’s potential difference resulting from oscillation, even with unknown coupling capacitance, the impedance of the object is measurable.
(3) By estimating the impedance of the measured object from the amplitude and phase spectrum obtained from the waveform of a few oscillation cycles, the temporal resolution of IS is improved.
(4) By making the oscillation waveform a non-sinusoidal wave, the fundamental frequency of oscillation and its higher harmonic waves are usable for the analysis. In this manner, the operation to switch frequency of a sinusoidal wave becomes unnecessary.

## 2. Approach of Capacitive-Coupling IS

### 2.1. Non-Sinusoidal Oscillator Circuit with Capacitive Couplings

**Figure 2:**
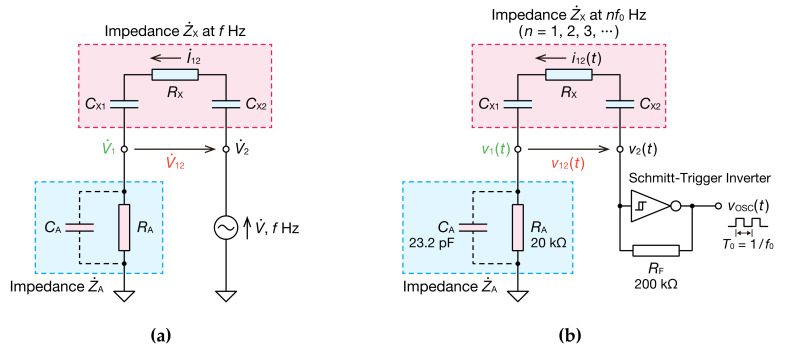
Circuit diagrams illustrating the measurement of impedance shown in Figure 1: (**a**) with a sinusoidal voltage source; and (**b**) in a non-sinusoidal oscillator.

Figure 2 illustrates circuit diagrams for the measurement of impedance shown in Figure 1.

In a circuit featuring the sinusoidal voltage of a single frequency (Figure 2a), an unknown impedance ${\dot{Z}}_{X}$ is obtained with Equation (1):

(1) $${\dot{Z}}_{X}=\frac{{\dot{V}}_{12}}{{\dot{I}}_{12}}=\frac{{\dot{V}}_{12}}{{\dot{V}}_{1}/{\dot{Z}}_{A}}=\frac{{\dot{V}}_{12}}{{\dot{V}}_{1}}\cdot {\dot{Z}}_{A}.$$

where ${\dot{V}}_{12}$ and ${\dot{I}}_{12}$ are the complex voltage and complex current of ${\dot{Z}}_{X}$, respectively, ${\dot{V}}_{1}$ is the complex voltage of ${\dot{Z}}_{A}$, and ${\dot{Z}}_{A}$ is the combined impedance of resistance $R_{A}$ and parasitic capacitance $C_{A}$. Based on this idea, in the present study, we obtained the frequency characteristics from the voltage signal of a non-sinusoidal oscillator. For the non-sinusoidal oscillator, including the capacitive coupling, we used a Schmitt trigger inverter oscillator. This oscillator does not have high precision for the oscillation frequency, but features a small number of parts and stable oscillation; thus, it makes for a small, cheap, and easy-to-handle system. Figure 2b shows the prepared oscillator. $R_{X}$ is a resistive element, which is the measured object, and $C_{X1}$ and $C_{X2}$ are the capacitive-coupling parts of the insulators and electrodes. Because ${\dot{Z}}_{X}=1/j\omega C_{X1}+R_{X}+1/j\omega C_{X2}=R_{X}+1/\left\{j\omega \left[C_{X1}C_{X2}/\left(C_{X1}+C_{X2}\right)\right]\right\}$, the capacitive couplings are considered to be incorporated into the oscillator as a capacitance $C_{X}=C_{X1}C_{X2}/\left(C_{X1}+C_{X2}\right)$. The resistive elements may also include a capacity component. However, in the present study, we assumed that there is only a resistive component for the sake of simplification. For the Schmitt trigger inverter integrated circuit (IC), we used a readily available 74HC14AP, and the power supply voltage of the IC was 5 V. The resistance for measuring the current $i_{12}\left(t\right)$ is $R_{A}$. Parasitic capacitance $C_{A}$ combines with $R_{A}$. The discharge and charge of the capacitor connected on the input side of the Schmitt trigger inverter switched the high and low levels of the voltage $v_{\mathrm{OSC}}\left(t\right)$, which led to the non-sinusoidal oscillation of the voltage $v_{1}\left(t\right)$ and $v_{2}\left(t\right)$. If parasitic capacitance $C_{A}$ can be ignored, the oscillation frequency $f_{0}$ can be expressed with the following:

(2) $$f_{0}=\frac{1}{\left(R_{F}+R_{A}+R_{X}\right)C_{X}\left(\ln\frac{V_{H}-V_{A}}{V_{H}-V_{P}}+\ln\frac{V_{B}-V_{L}}{V_{N}-V_{L}}\right)},$$

(3) $$V_{A}=V_{N}+\frac{R_{A}+R_{X}}{R_{F}+R_{A}+R_{X}}\left(V_{H}-V_{L}\right),$$

(4) $$V_{B}=V_{P}-\frac{R_{A}+R_{X}}{R_{F}+R_{A}+R_{X}}\left(V_{H}-V_{L}\right).$$

where $V_{H}$ is 5 V for the high-level output and 0 V for the low-level output of the Schmitt trigger inverter. In addition, $V_{P}$ and $V_{N}$ are the threshold voltages of the Schmitt trigger inverter, where $V_{P}>V_{N}$. Equation (2) shows that $f_{0}$ is determined by the $R_{X}$ and $C_{X}$ of the capacitive-coupling part. Even if $f_{0}$ is determined, the satisfactory combination of $R_{X}$ and $C_{X}$ is infinite; thus, Equation (2) alone cannot uniquely determine $R_{X}$ and $C_{X}$.

### 2.2. Determination of Unknown Capacitance and Resistance in Series Connection

With a normal IS, sine waves with varying frequencies are input into the unknown impedance, and the impedance characteristics are measured and calculated. For example, in Figure 2a, the impedance ${\dot{Z}}_{X}\left(f\right)$ at the frequency of f can be calculated with the following equation:

(5) $${\dot{Z}}_{X}\left(f\right)=\frac{\left|{\dot{V}}_{12}\left(f\right)\right|}{\left|{\dot{V}}_{1}\left(f\right)\right|}\cdot \left|{\dot{Z}}_{A}\left(f\right)\right|e^{j\left\{\theta_{V12}\left(f\right)-\theta_{V1}\left(f\right)+\theta_{\mathrm{ZA}}\left(f\right)\right\}}.$$

$\mathrm{where}\;\left|{\dot{V}}_{12}\left(f\right)\right|$ and $\left|{\dot{V}}_{1}\left(f\right)\right|$ are the measured amplitude of ${\dot{V}}_{12}\left(f\right),$ ${\dot{V}}_{1}\left(f\right)$ and the $\theta_{V12}\left(f\right)$, $\theta_{V1}\left(f\right)$ phase. The absolute value $\left|{\dot{Z}}_{A}\left(f\right)\right|$ and phase $\theta_{\mathrm{ZA}}\left(f\right)$ of ${\dot{Z}}_{A}\left(f\right)$ are theoretically derived. By obtaining frequency–gain characteristics (amplitude spectrum) and frequency–phase characteristics (phase spectrum), IS can be actualized.

If ${\dot{Z}}_{X}\left(f\right)$ was incorporated into part of the non-sinusoidal oscillator (Figure 2b), the measured $v_{12}\left(t\right)$ and $v_{1}\left(t\right)$ have periodic waveforms of non-sinusoidal waves with an oscillation frequency $f_{0}$. Therefore, the Fourier series expansion of one cycle, $T_{0}=1/f_{0}$, of the observed waveforms can be expressed with Equations (6)–(9) using the complex Fourier coefficients ${\dot{V}}_{12}^{\prime }\left(kf_{0}\right)$ and ${\dot{V}}_{1}^{\prime }\left(kf_{0}\right)$:

(6) $$v_{12}\left(t\right)=\overset{\infty }{\underset{k=-\infty }{\sum }}{\dot{V}}_{12}^{\prime }\left(kf_{0}\right)e^{j2\pi kf_{0}t},$$

(7) $${\dot{V}}_{12}^{\prime }\left(kf_{0}\right)=\frac{1}{T_{0}}\int_{0}^{T_{0}}v_{12}\left(t\right)e^{-j2\pi kf_{0}t}dt,$$

(8) $$v_{1}\left(t\right)=\overset{\infty }{\underset{k=-\infty }{\sum }}{\dot{V}}_{1}^{\prime }\left(kf_{0}\right)e^{j2\pi kf_{0}t},$$

(9) $${\dot{V}}_{1}^{\prime }\left(kf_{0}\right)=\frac{1}{T_{0}}\int_{0}^{T_{0}}v_{1}\left(t\right)e^{-j2\pi kf_{0}t}dt.$$

Therefore, we can assume that $v_{12}\left(t\right)$ and $v_{1}\left(t\right)$ consist of sine waves with a fundamental frequency $f_{0}$ and its integer multiple $kf_{0}$. Each frequency component’s coefficient comes from the DFT of one cycle as an amplitude spectrum $\left|{\dot{V}}_{12}\left(kf_{0}\right)\right|$ and $\left|{\dot{V}}_{1}\left(kf_{0}\right)\right|$ and phase spectrum $\theta_{V12}\left(kf_{0}\right)$ and $\theta_{V1}\left(kf_{0}\right)$. In Equations (6) and (8), the negative frequency component (k<0) is a complex conjugate of the positive frequency component:

(10) $$\left|{\dot{V}}_{12}\left(kf_{0}\right)\right|=2\left|{\dot{V}}_{12}^{\prime }\left(kf_{0}\right)\right|,$$

(11) $$\left|{\dot{V}}_{1}\left(kf_{0}\right)\right|=2\left|{\dot{V}}_{1}^{\prime }\left(kf_{0}\right)\right|.$$

If $v_{1}\left(t+T_{0}/2\right)=-v_{1}\left(t\right)$, $v_{12}\left(t\right)$ and $v_{1}\left(t\right)$ only include the odd-order frequency component.

Furthermore, the impedance ${\dot{Z}}_{A}\left(f\right)=\left|{\dot{Z}}_{A}\left(f\right)\right|e^{j\theta_{\mathrm{ZA}}\left(f\right)}$ can be expressed with the following equation as a continuous function of f:

(12) $$\left|{\dot{Z}}_{A}\left(f\right)\right|=\frac{1}{\sqrt{{\left(\frac{1}{R_{A}}\right)}^{2}+{\left(2\pi fC_{A}\right)}^{2}}},$$

(13) $$\theta_{\mathrm{ZA}}\left(f\right)=-\tan^{-1}\left(2\pi fC_{A}R_{A}\right).$$

Therefore, for each frequency of $nf_{0}$ (n=1,2,3,⋯), amplitude and phase spectrums,

(14) $$\left|{\dot{Z}}_{A}\left(nf_{0}\right)\right|=\frac{1}{\sqrt{{\left(\frac{1}{R_{A}}\right)}^{2}+{\left(2\pi nf_{0}C_{A}\right)}^{2}}},$$

(15) $$\theta_{\mathrm{ZA}}\left(nf_{0}\right)=-\tan^{-1}\left(2\pi nf_{0}C_{A}R_{A}\right)$$

are obtained. Thus, the amplitude and phase spectrum, $\left|{\dot{Z}}_{X}\left(nf_{0}\right)\right|$ and $\theta_{Z12}\left(nf_{0}\right)$, of unknown impedance ${\dot{Z}}_{X}$ are obtained with Equations (16) and (17) using the amplitude and phase spectra of ${\dot{V}}_{12}$, ${\dot{V}}_{1}$, and ${\dot{Z}}_{A}$:

(16) $$\left|{\dot{Z}}_{X}\left(nf_{0}\right)\right|=\frac{\left|{\dot{V}}_{12}\left(nf_{0}\right)\right|}{\left|{\dot{V}}_{1}\left(nf_{0}\right)\right|}\cdot \left|{\dot{Z}}_{A}\left(nf_{0}\right)\right|,$$

(17) $$\theta_{Z12}\left(nf_{0}\right)=\theta_{V12}\left(nf_{0}\right)-\theta_{V1}\left(nf_{0}\right)+\theta_{\mathrm{ZA}}\left(nf_{0}\right).$$

Furthermore, with Equations (16) and (17), the frequency response for the real and imaginary parts of impedance ${\dot{Z}}_{X}$, Re(Z˙X) and Im(Z˙X), are obtained with Equations (18) and (19):

(18) Re(Z˙X)=V˙12nf0V˙1nf0·Z˙Anf0cosθV12nf0−θV1nf0+θZAnf0,

(19) Im(Z˙X)=V˙12nf0V˙1nf0·Z˙Anf0sinθV12nf0−θV1nf0+θZAnf0.

The frequency response of the real and imaginary parts of admittance ${\dot{Y}}_{X}$, Re(Y˙X) and Im(Y˙X), can be obtained with Equations (20) and (21) by setting ${\dot{Y}}_{X}\left(nf_{0}\right)=1/{\dot{Z}}_{X}\left(nf_{0}\right)$:

(20) Re(Y˙X)=1Z˙Xnf0cosθZ12nf0,

(21) Im(Y˙X)=−1Z˙Xnf0sinθZ12nf0.

Next, if we know that ${\dot{Z}}_{X}$ is a resistive element and capacitive coupling in series, as in Figure 1b, the frequency responses of the real and imaginary parts of the impedance are:

(22) Re(Z˙X)=RX,

(23) Im(Z˙X)=−12πnf0CX.

Thus, $R_{X}$ and $C_{X}$ can be estimated by applying the least-squares method to the finite number of spectra of frequency, $nf_{0}$ (n=1, 2, 3, ⋯, N).

### 2.3. Experimental Method

For the oscillator in Figure 2b, we used multiple high-precision metal film resistors and one multilayer ceramic capacitor to mimic the resistive element $R_{X}$ and the capacitive coupling $C_{X}$. The $R_{X}$ was changed between 0.20 and 10.0 kΩ using the series connection of resistance of 0.10 or 1.0 kΩ (tolerance of ±0.1% for both). The nominal values of the $C_{X}$ were 0.10 nF, 1.0 nF (tolerance of ±5% for both), or 10 nF (tolerance of ±10%). The $R_{X}$ and $C_{X}$ were wired on a breadboard.

The time waveform data of the voltage were collected using a digital oscilloscope (OWON VDS3104L). $v_{1}\left(t\right)$ and $v_{2}\left(t\right)$ in Figure 2b were measured, and the time waveform data of $v_{12}\left(t\right)$ was obtained from their difference. Then, we transformed DFT for two cycles ($C_{X}$ = 1.0 nF or 10 nF) or four cycles ($C_{X}$ = 0.10 nF) of the time waveform data. The time waveform data was divided by a certain number of data points, and DFT was performed for each cycle. We averaged each DFT result. For the window function of DFT, we used a rectangular window. From the obtained DFT data, we obtained real and imaginary parts for ${\dot{Z}}_{X}$ following the steps discussed in Section 2.2. $R_{X}$ and $C_{X}$ were estimated using the least-squares method.

## 3. Results

**Figure 3:**
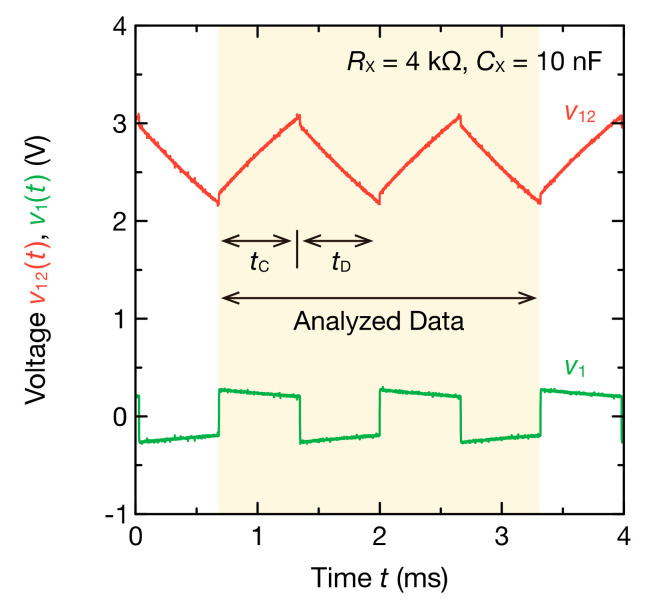
Example of the oscillation waveforms of $v_{12}\left(t\right)$ and $v1\left(t\right)$ measured in the circuit of Figure 2b. The $R_{X}$ and $C_{X}$ were set to 4.0 kΩ and 10 nF, respectively. The segment of time with a colored background corresponds to two cycles of the oscillation and was used for the subsequent discrete Fourier transform (DFT) analysis.

**Figure 4:**
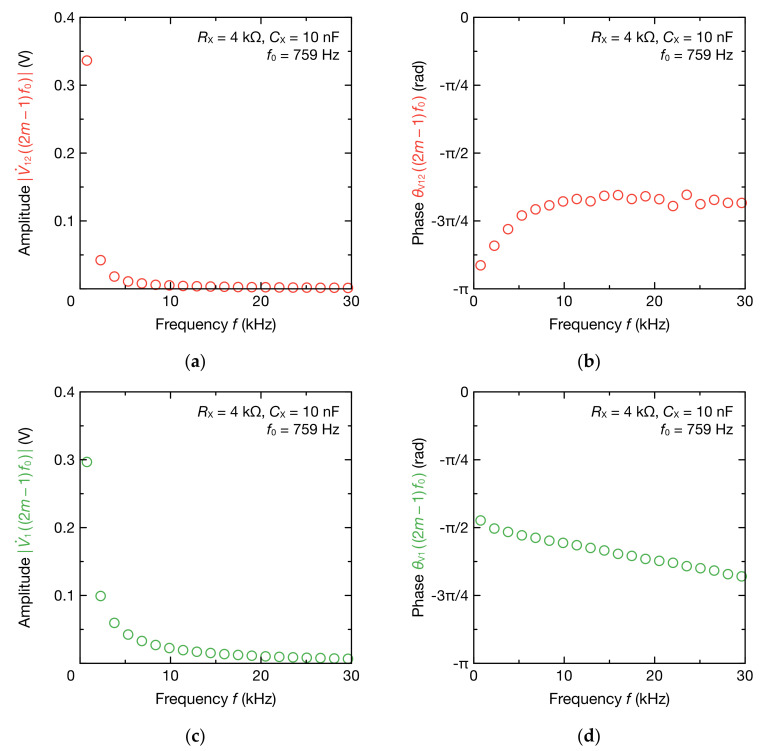
Frequency spectra of: (**a**) amplitude $\left|{\dot{V}}_{12}\right|$; (**b**) phase $\theta_{V12}$; (**c**) amplitude $\left|{\dot{V}}_{1}\right|$; and (**d**) phase $\theta_{V1}$ at $\left(2m-1\right)f_{0}$ (m=1, 2, 3, ⋯, 20 ) Hz. The spectra were obtained using DFT from the two-cycle segment of $v_{12}\left(t\right)$ and $v_{1}\left(t\right)$ in Figure 3. The $R_{X}$ and $C_{X}$ were set to 4.0 kΩ and 10 nF, respectively.

The oscillation waveforms for the circuit in Figure 2b are shown in Figure 3. The frequency spectra of ${\dot{V}}_{12}$ and ${\dot{V}}_{1}$ were obtained from the DFT of these waveforms (Figure 4). The $R_{X}$ and $C_{X}$ were 4.0 kΩ and 10 nF, respectively. When $C_{X}$ was charged and $v_{12}\left(t\right)$ increased to about 3.1 V, the output voltage of the Schmitt trigger inverter $v_{\mathrm{OSC}}\left(t\right)$ switched from $V_{H}$ to $V_{L}$. This caused a discharge of $C_{X}$ and $v_{12}\left(t\right)$ was reduced. When $v_{2}\left(t\right)$ decreased to about 2.2 V, $v_{\mathrm{OSC}}\left(t\right)$ was switched from $V_{L}$ to $V_{H}$, and $C_{X}$ was charged again. This discharge and the charge of $C_{X}$ created oscillation. The time it took to charge $C_{X}$, $t_{C}$, was 0.663 ms. The time it took to discharge $t_{D}$ was 0.654 ms. Therefore, data for the two cycles were collected in 2.6 ms, enabling analysis in a short period, even when the DFT calculation time was included. Furthermore, $t_{C}\approx t_{D}$ and $v_{1}\left(t+T_{0}/2\right)=-v_{1}\left(t\right)$ were mostly satisfied; thus, the frequency components included in the oscillation waveform were odd harmonics $\left(2m-1\right)f_{0}$(m=1, 2, 3, ⋯).

Next, Figure 4 shows odd harmonic components of ${\dot{V}}_{12}$ and ${\dot{V}}_{1}$ to the 39th order (N=39). Both ${\dot{V}}_{12}\left(\left(2m-1\right)f_{0}\right)$ and ${\dot{V}}_{1}\left(\left(2m-1\right)f_{0}\right)$ contained odd harmonic components with a fundamental frequency of 759 Hz (Figure 4a,c). Because $v_{12}\left(t\right)$ was a waveform close to a triangular wave, the amplitude of ${\dot{V}}_{12}\left(\left(2m-1\right)f_{0}\right)$ was inversely proportional to the square of the harmonic order. By comparison, because $v_{1}\left(t\right)$ is a waveform close to a square wave, the amplitude of ${\dot{V}}_{1}\left(\left(2m-1\right)f_{0}\right)$ was inversely proportional to the harmonic order. Furthermore, the phase of ${\dot{V}}_{12}\left(\left(2m-1\right)f_{0}\right)$ increased as the frequency increased from −2.87 rad, whereas the phase of ${\dot{V}}_{1}\left(\left(2m-1\right)f_{0}\right)$ decreased as the frequency increased from −1.49 rad (Figure 4b,d).

**Figure 5:**
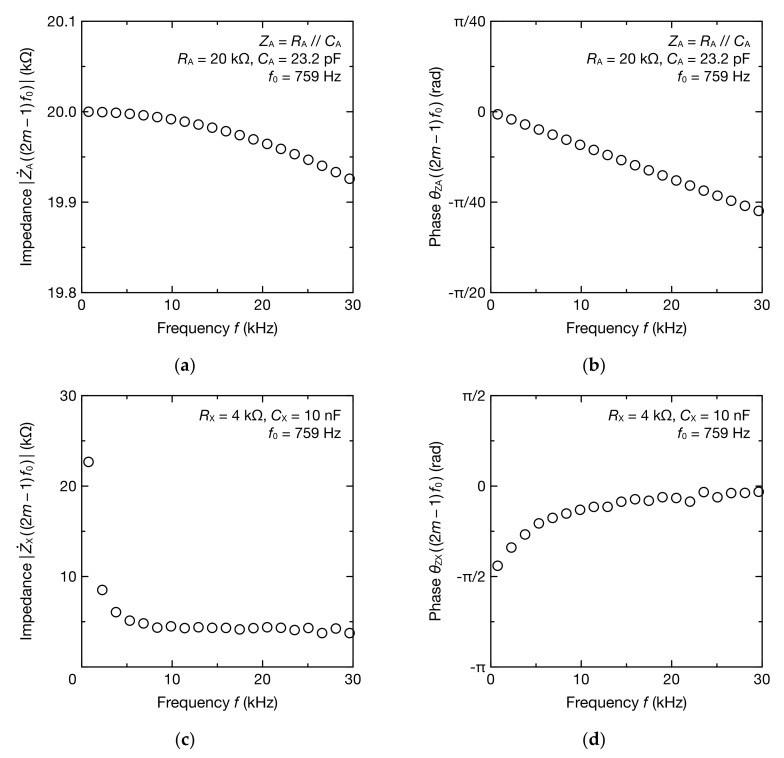
Frequency spectra of: (**a**) absolute impedance $\left|{\dot{Z}}_{A}\right|$; (**b**) phase $\theta_{\mathrm{ZA}}$; (**c**) absolute impedance $\left|{\dot{Z}}_{X}\right|$; and (**d**) phase $\theta_{\mathrm{ZX}}$ at $\left(2m-1\right)f_{0}$ (m=1, 2, 3, ⋯, 20 ) Hz.

The result of estimating the impedance ${\dot{Z}}_{X}$ from the obtained frequency spectrum of ${\dot{V}}_{12}$ and ${\dot{V}}_{1}$ using Equations (16) and (17) is shown in Figure 5. The absolute value and phase of ${\dot{Z}}_{A}$ necessary to estimate ${\dot{Z}}_{X}$ were obtained from Equations (14) and (15) (Figure 5a,b). As the frequency increased, the absolute value of the estimated ${\dot{Z}}_{X}$, $\left|{\dot{Z}}_{X}\right|$, decreased, whereas phase $\theta_{\mathrm{ZX}}$ gradually increased from −1.38 rad to −0.0988 rad (Figure 5c,d). This reflects the frequency characteristics of resistor-capacitor (RC) series circuits.

**Figure 6:**
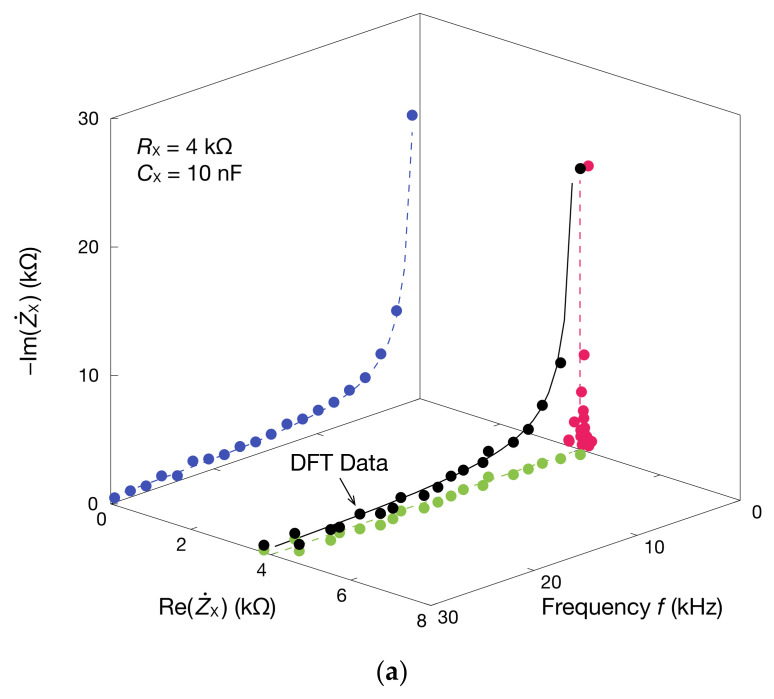

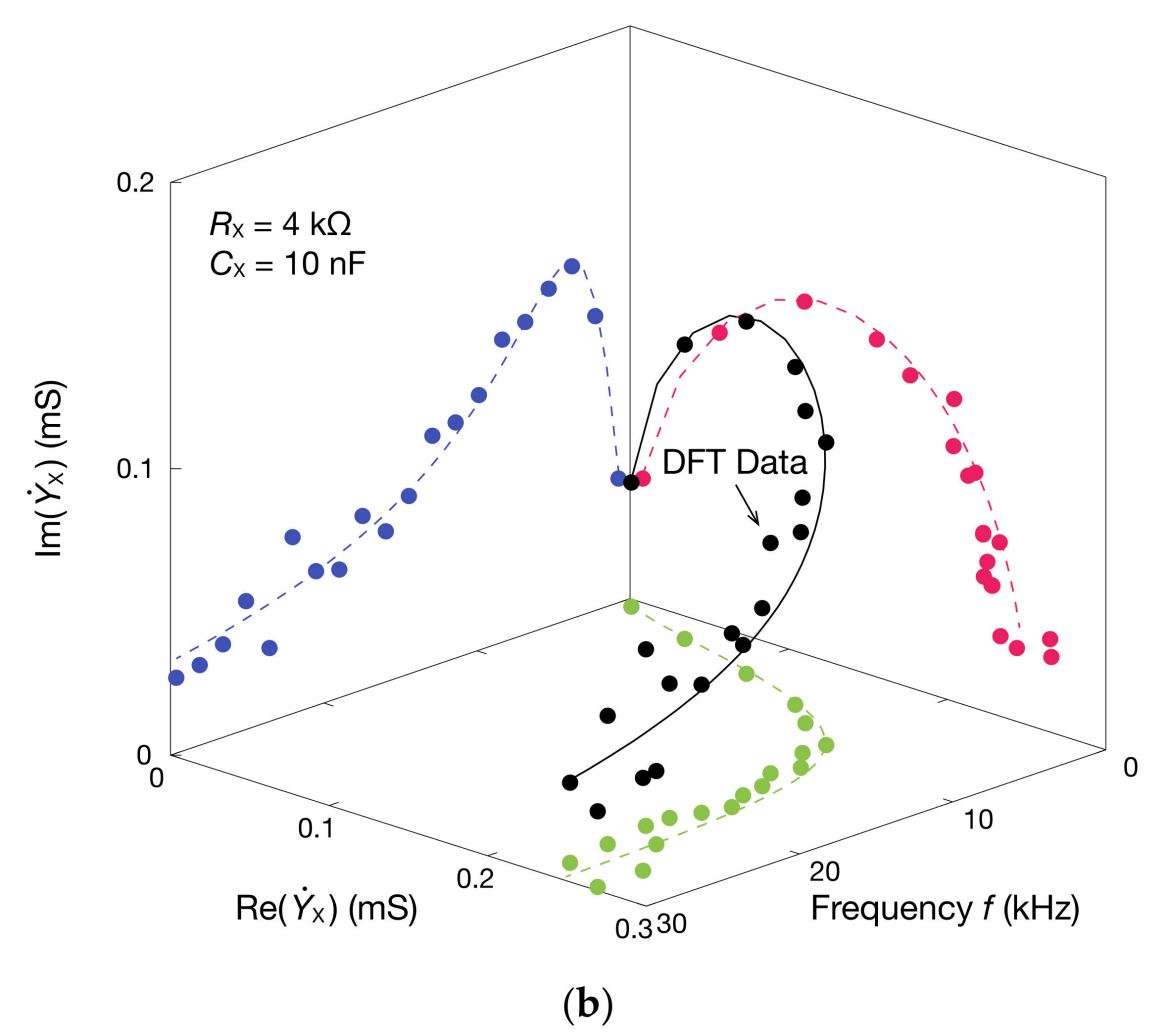
Three-dimensional perspective plots of: (**a**) impedance ${\dot{Z}}_{X}$; and (**b**) admittance ${\dot{Y}}_{X}$. Three-dimensional DFT data is projected onto each plane. The solid and dashed lines are theoretical curves of ${\dot{Z}}_{X}$ and ${\dot{Y}}_{X}$.

**Figure 7:**
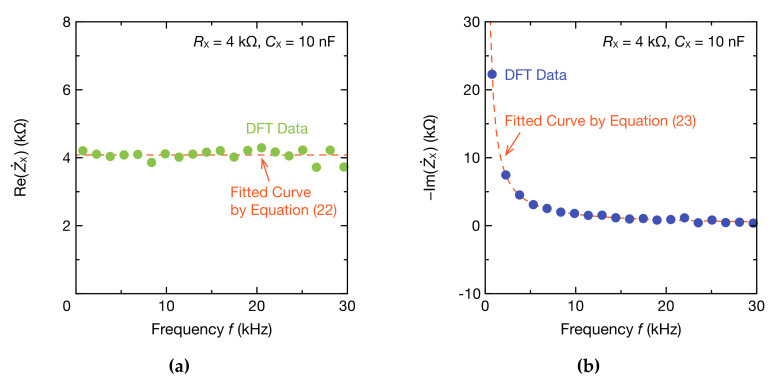
Frequency spectra of: (**a**) real part Re(Z˙X); and (**b**) imaginary part Im(Z˙X) of impedance ${\dot{Z}}_{X}$. The DFT data were fitted to Equations (22) and (23) for determining $R_{X}$ and $C_{X}$ (dashed lines).

In addition, the real and imaginary parts of ${\dot{Z}}_{X}$ and ${\dot{Y}}_{X}$ were obtained from the absolute value and phase of ${\dot{Z}}_{X}$ using Equations (18) and (21). A 3D graph with the frequency is shown in Figure 6 [58]. Because $R_{X}$ and $C_{X}$ are connected in series, the real part of ${\dot{Z}}_{X}$, Re(Z˙X), equals $R_{X}$, and remains constant regardless of the frequency. The imaginary part of ${\dot{Z}}_{X}$, Im$\left({\dot{Z}}_{X}\right)$, is inversely proportional to the frequency. The Cole–Cole plot of ${\dot{Z}}_{X}$ is a straight line in the case of impedance and a semicircle in that of admittance. It showed that the impedance spectrum necessary to analyze EIS and BIS could be obtained using the proposed method. Estimates of the resistance and capacitance are shown in Figure 7. Using the data from the real part of ${\dot{Z}}_{X}$, the resistance $R_{X}$ was estimated with Equation (22), and the result was 4.082 kΩ. Furthermore, the capacitance $C_{X}$ can be estimated from the imaginary part of ${\dot{Z}}_{X}$, corresponding to Equation (23), which led to 9.395 nF. The errors between these estimates and the nominal values were 2.05% for $R_{X}$ and −6.05% for $C_{X}$. The estimation error of the $C_{X}$ is within the tolerance of the used capacitor and is considered a reasonable level of accuracy. As such, the proposed method can estimate the resistance of the resistive element and coupled capacitance.

**Figure 8:**
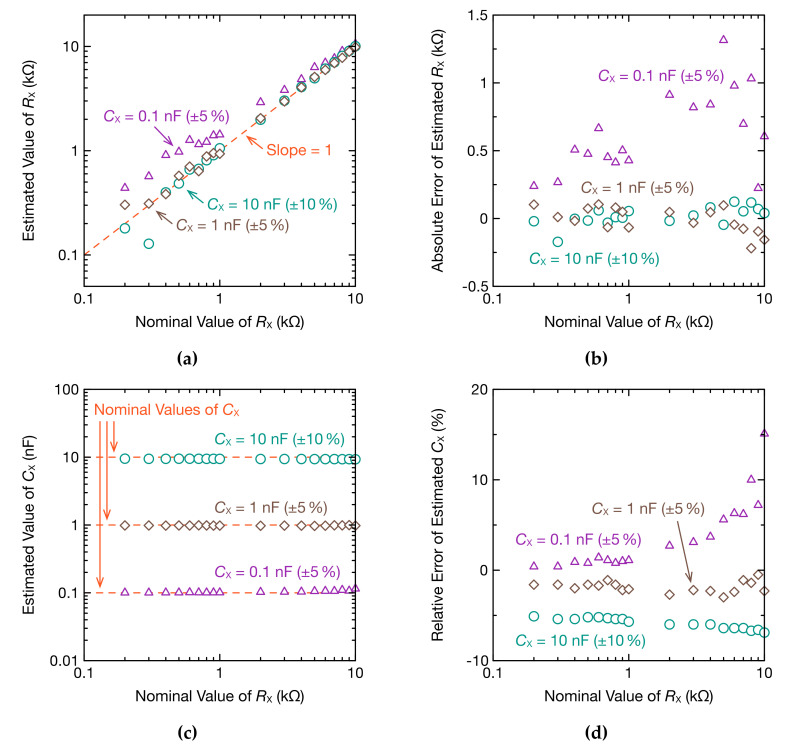
$R_{X}$ and $C_{X}$ estimated using oscillation waveforms and DFT: (**a**) estimated $R_{X}$; (**b**) absolute error of estimated $R_{X}$; (**c**) estimated $C_{X}$; (**d**) relative error of estimated $C_{X}$.

We also examined whether the resistance and capacitance can be estimated when $C_{X}$ was lowered to 1.0 or 0.10 nF. For an $R_{X}$ from 0.20 to 10.0 kΩ, and $C_{X}$ at 10, 1.0, and 0.10 nF, the resistance and capacitance were estimated (Figure 8). When $C_{X}$ was 10 or 1.0 nF, the error between the resistance and capacitance, and their respective nominal values, was less than 5% in the range of $R_{X}$ from 2.0 to 10.0 kΩ, which is a highly precise estimate. Although the precision of the resistance estimate decreased in the range of $R_{X}$ from 0.40 to 1.0 kΩ, estimation was still possible. The impedance of a living body is generally several hundred Ω, and so the present method can also be applied to BIS. However, when $C_{X}$ was 0.10 nF, the relative error of estimated low-resistance values was high, making estimations difficult.

## 4. Discussion

With the proposed method, we could estimate $R_{X}$ in the range of 0.90 to 10.0 kΩ and $C_{X}$ of 10 or 1.0 nF with accuracy. If $R_{X}$ is smaller than 0.90 kΩ or $C_{X}$ is 0.10 nF, the estimate was less precise. This likely has an impact on the parasitic impedance ${\dot{Z}}_{A}$ in wires and circuit boards, in addition to the $R_{A}$ and $C_{A}$. Thus, to improve the precision for low resistance and capacitance, we determined a more detailed equivalent circuit model of ${\dot{Z}}_{A}$ from the Cole–Cole plot of ${\dot{Z}}_{A}$ when $C_{X}=$ 0.10 nF and $R_{X}=$ 0 Ω. When $C_{X}$ was known, ${\dot{Z}}_{A}$ was obtained with ${\dot{V}}_{12}\left(nf_{0}\right)$ and ${\dot{V}}_{1}\left(nf_{0}\right)$ with DFT using Equation (24):

(24) $${\dot{Z}}_{A}\left(nf_{0}\right)=\frac{\left|{\dot{V}}_{1}\left(nf_{0}\right)\right|}{\left|{\dot{V}}_{12}\left(nf_{0}\right)\right|}\cdot \frac{1}{2\pi nf_{0}C_{X}}e^{j\left\{\theta_{V1}\left(nf_{0}\right)-\theta_{V12}\left(nf_{0}\right)-\frac{\pi }{2}\right\}}.$$

**Figure 9:**
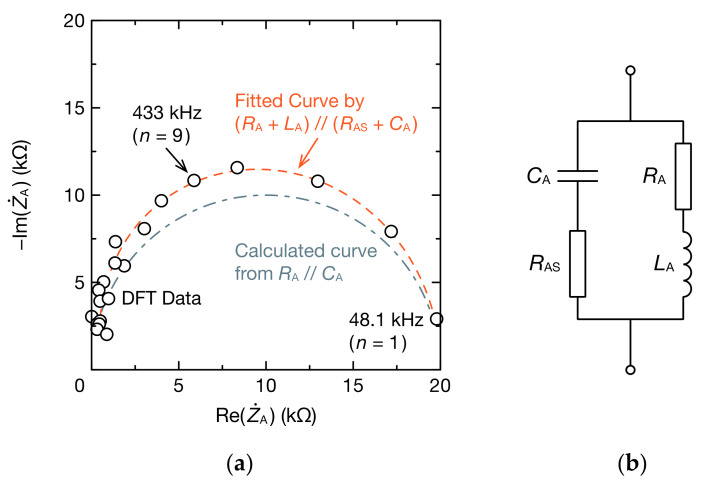
Equivalent circuit modeling of ${\dot{Z}}_{A}$: (**a**) Cole–Cole plot for $C_{X}=$ 0.10 nF and $R_{X}=$ 0 Ω; (**b**) equivalent circuit with a stray resistance $R_{\mathrm{AS}}$ and stray inductance $L_{A}$. The symbol n represents the harmonic number of ${\dot{Z}}_{A}\left(nf_{0}\right)$. We used pyZwx software to fit the DFT data to the equivalent circuit model [59].

Figure 9a shows the Cole–Cole plot of ${\dot{Z}}_{A}$. The DFT data creates a capacitive semicircle and is under the notable impact of $R_{A}$ and $C_{A}$. However, there is a discrepancy between a curve calculated from $R_{A}=$ 20.0 kΩ and $C_{A}=$ 23.2 pF only ($R_{A}//C_{A}$) and DFT data, indicating that there is parasitic impedance other than $R_{A}$ and $C_{A}$ in ${\dot{Z}}_{A}$. As in Figure 9b, when a curve fitting was performed for an equivalent circuit model that considers parasitic resistance $R_{\mathrm{AS}}$ and parasitic inductance $L_{A}$, in addition to $R_{A}$ and $C_{A}$ ($\left(R_{A}+L_{A}\right)//\left(R_{\mathrm{AS}}+C_{A}\right)$), the result was consistent with the DFT data. Each parameter of the equivalent circuit was $R_{A}=$ 20.017 kΩ, $R_{\mathrm{AS}}=$ 378.761 Ω, $L_{A}=$ 4.705 mH and $C_{A}=$ 35.416 pF. For $R_{\mathrm{AS}}$, there are possibilities of resistance from the wiring pattern of the circuit board and contact resistance, but because $L_{A}$ is too large as a parasitic inductance, a separate interpretation of $L_{A}$ is needed.

**Figure 10:**
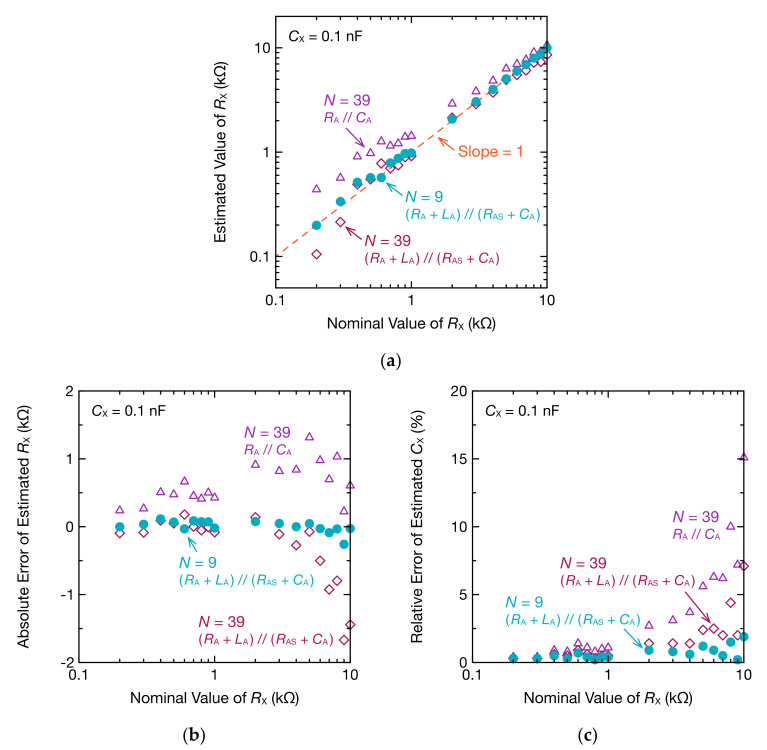
$R_{X}$ and $C_{X}$ estimated using optimized ${\dot{Z}}_{A}$: (**a**) estimated $R_{X}$; (**b**) absolute error of estimated $R_{X}$; (**c**) relative error of estimated $C_{X}$.

Next, using the obtained circuit parameters, $R_{X}$ and $C_{X}$ were estimated again (Figure 10). When estimated from odd harmonics to the 39th order, by considering both $R_{\mathrm{AS}}$ and $L_{A}$, the precision of the low-resistance estimate was improved. However, there was less improvement in high-resistance values (Figure 10a,b). A similar trend was observed in the estimates of $C_{X}$(Figure 10c). This indicates that, at higher frequencies, the parasitic capacitance of $R_{X}$ and a breadboard cannot be ignored. Therefore, we limited the frequency for the estimate to odd harmonics of up to 500 kHz (N= 9) and found that the estimate precision was also improved in higher resistance values. When $C_{X}$ was 10 or 1.0 nF, the precision did not change, even taking $R_{\mathrm{AS}}$ and $L_{A}$ into consideration. Therefore, by optimizing the equivalent circuit model of ${\dot{Z}}_{A}$ and keeping the frequency of the higher harmonic wave to 500 kHz or below, estimates of low resistance and low capacitance could be improved. When the frequency of higher harmonic waves is limited, the number of data points for fitting is low when the oscillation frequency is high, which is a problem when estimating a complex circuit’s impedance. However, because Schmitt trigger oscillators can change the oscillation frequency with $R_{F}$, measurements with multiple $R_{F}$ enable a sufficient number of data points.

## 5. Conclusions

In this study, we proposed a new capacitive-coupling IS that uses a non-sinusoidal oscillator and DFT. To verify this method, we prepared a Schmitt trigger oscillator that incorporated a measured object and a capacitive coupling. This circuit is extremely simple and can be miniaturized. Furthermore, the oscillator’s non-sinusoidal voltage waveform can be DFT-transformed to obtain the fundamental frequency and odd harmonic components. The resistance and capacitance that mimic a resistive element and capacitive-coupling component can be estimated by obtaining impedance from the amplitude of the voltage and phase spectrum. Furthermore, when the coupled capacitance is small, there is more impact from parasitic impedance, such as from circuit boards. This is a factor that reduces the precision of the estimate, but by correcting for parasitic impedance and limiting the order of the higher harmonic waves used for estimates, the estimation precision is improved.

For the practical application of our proposed system, we must analyze more complex circuits consisting of several series or parallel RC circuits. To improve the accuracy of the regression analysis, more DFT data points at low frequencies are necessary to accurately estimate the resistance and capacitance of complex circuits. Although we fixed the resistance $R_{F}$ of the fabricated oscillation circuit at 200 kΩ, this can also easily switch to higher resistance. Therefore, we can acquire non-sinusoidal waveform data quickly at lower oscillation frequencies, which enables more DFT data points to be obtained.

In the future, we will apply the present system to measuring the impedance of various targets, such as a living body, and coating delamination and corrosion processes.
